# Administration of Beta-Nerve Growth Factor during the Preovulatory Stage Improves Endocrine and Luteal Function in Dairy Heifers

**DOI:** 10.3390/ani13061004

**Published:** 2023-03-09

**Authors:** Gonzalo Gajardo, Luis Paiva, Cesar Ulloa-Leal, Ximena Valderrama, Gerardo López, Albert Carrasco, Alejandra Isabel Hidalgo, Mauricio E. Silva, Patricio I. Palma, Marcelo H. Ratto

**Affiliations:** 1 Escuela de Graduados, Facultad de Ciencias Veterinarias, Universidad Austral de Chile, Valdivia 5091000, Chile; 2 Escuela de Medicina Veterinaria, Facultad de Agronomía e Ingeniería Forestal, Facultad de Ciencias Biológicas y Facultad de Medicina, Pontificia Universidad Católica de Chile, Santiago 7820436, Chile; 3 Instituto de Ciencia Animal, Facultad de Ciencias Veterinarias, Universidad Austral de Chile, Valdivia 5091000, Chile; 4 Escuela de Medicina Veterinaria, Facultad de Ciencias Veterinarias, Universidad de Concepción, Chillán 3812120, Chile; 5 Departamento de Medicina Veterinaria y Salud Publica, Facultad de Recursos Naturales, Universidad Católica de Temuco, Temuco 4781312, Chile; 6 Lecherías del Sur SpA, Francisco Bilbao 1860, Osorno 5310644, Chile

**Keywords:** neurotrophin, pregnancy, bovine, reproductive biotechnology, artificial insemination

## Abstract

**Simple Summary:**

Certain proteins present in semen regulate the reproductive physiology of females. Beta-nerve growth factor (NGF), which is abundantly expressed in the semen of camelids, has a potent ovulatory and luteotrophic effect in llamas after systemic injection. Here, we investigated the effects of intramuscular NGF during the preovulatory stage on endocrine parameters and pregnancy rates after artificial insemination in dairy heifers. Heifers injected with NGF showed enhancement of hormones related to ovulation and gestation maintenance. We also detected the enhancement of genes related to uterine receptivity and higher pregnancy rates. We conclude that the improvement in reproductive hormones related to gestation is possibly linked to the higher pregnancy rate detected here. More research is needed to ensure the NGF effects remain on commercial exploitation farms.

**Abstract:**

The neurotrophin beta-nerve growth factor (NGF), which is present in the semen of different mammals, elicits potent ovulatory and luteotrophic actions in llamas following systemic administration. Here, we determine if purified NGF given intramuscularly (IM) during the preovulatory stage affects the corpus luteum (CL), hormone production, endometrial gene expression, and pregnancy rate of dairy heifers. Holstein-Friesian heifers were estrus-synchronized using estradiol benzoate (EB) plus an intravaginal progesterone (P4) device (DIB). After eight days, the device was removed and cloprostenol was given IM; the next day (day 9), heifers received EB IM plus one of the following: (i) 1 mg of NGF (NGF D9 group), (ii) 1 mg of NGF 32 h after EB (NGF D10 group), or (iii) phosphate buffer saline (control group). To measure pregnancy rates, heifers were treated similarly, then artificially inseminated with sexed semen 48–52 h after DIB removal, then an ultrasound was conducted 30 days after insemination. The females given NGF along with EB (NGF D9) showed significantly higher luteinizing hormone (LH) concentrations, larger CL vascular areas, and higher plasma P4 concentrations than the NGF D10 and control animals. Downregulation of the P4 receptor (*PGR*), and upregulation of both lipoprotein lipase (*LPL*) and Solute Carrier Family 6 member 14 (*SLC6A14*) endometrial genes, were detected in NGF D9 heifers. Furthermore, these heifers had a 10% higher pregnancy rate than the control group. We conclude that the higher P4 output, in response to the early NGF administration, led to the enhanced gene expression of transcripts related to uterine receptivity that may result in enhanced pregnancy rates.

## 1. Introduction

The neurotrophin beta-nerve growth factor (NGF) present in the ejaculate of camelids is known to influence the reproductive physiology of llamas and alpacas [1,2]. Several studies [3] show that, in llamas, NGF given by intramuscular (IM), intravenous (IV), or intrauterine routes robustly induces ovulation and has a potent luteotrophic effect, since the corpora lutea (CL) induced by this protein produces more progesterone (P4) than that induced by the gonadotropin-releasing hormone (GnRH).

Although the detection of NGF in the ejaculates of spontaneous ovulatory species was reported many years ago [4,5], its potential modulatory effects on reproduction are still not well understood. A study [6] reported that IM administration of 2 mL of raw bovine seminal plasma induced ovulation in 5/19 (26%) llamas, suggesting that bovine semen contains factors capable of modulating reproductive function in females. Furthermore, Tanco et al. [7] showed that purified NGF (1 mg/100 kg IM) failed to induce ovulation in both prepubertal and mature beef heifers; however, heifers treated with NGF on day 5 after ovulation exhibited higher P4 concentrations than those given GnRH and bearing an accessory corpus luteum (CL), indicating a luteotrophic role of this neurotrophin on luteal function. Consistently, early administration of 250 µg of NGF from bovine seminal plasma at 4 h after ovulation enhances P4 production in beef heifers [8].

In one bovine study [9], Angus cows were estrus-synchronized, artificially inseminated (AI) with frozen semen, then given 296 µg of purified bovine NGF from seminal plasma; consequently, they exhibited a significant increase in plasma P4 concentrations from day 10 to day 19 after AI compared to non-treated cows. There was also a significant increase in the expression of interferon-stimulated genes, including *ISG15* and *MX2*, with a 16% higher pregnancy rate than non-treated cows. In another recent study [10], the IM administration of 1 mg of llama NGF, given at the estradiol benzoate (EB)-induced luteinizing hormone (LH) surge, resulted in a significant increase in CL vascular area and higher P4 concentrations on days 6, 9, and 15 after ovulation in dairy heifers. However, in estrus-synchronized lactating Holstein dairy cows, the administration of 296 µg of bull NGF did not affect pregnancy rates, nor embryonic mortality, compared to untreated cows [11].

The luteotrophic effects of NGF have been linked to an increase in serum LH and P4 concentration, when administered together with a GnRH analogue in an estrous synchronization protocol, as shown by Stewart et al. [12]. The study reported a significant increase in the total number of small luteal cells, upregulation of the steroidogenic acute regulatory protein (*STAR*) gene, and mild upregulation of the LH receptor (*LHR*) and cytochrome P450 family 11 (*CYP11A1*) genes. Similar effects have been described in llamas [13,14].

In the present study, we investigate the effects of systemic administration of heterologous (llama) NGF during the preovulatory stage of dairy heifers synchronized with an EB/P4-based, timed AI protocol on LH, estradiol (E2), P4 blood content, and CL function. Furthermore, we investigate whether the administration of this protein enhances genes related to uterine receptivity and pregnancy rates on a commercial dairy farm.

## 2. Materials and Methods

Animals were maintained in facilities of the Universidad Austral de Chile (UACH), Valdivia, Chile, under university animal care regulations. Experimental procedures were reviewed and approved (ref. 351/2019) by the University Bioethical Committee and were performed in accordance with the Chilean Animal Protection Act (2009).

### 2.1. NGF Isolation and Purification

The beta-NGF was obtained from llama seminal plasma and purified as previously described [1,15]. Briefly, semen was collected twice per week from three mature male llamas, then each ejaculate was diluted 1:1 (vol/vol) with phosphate-buffered saline (PBS) and centrifuged for 30 min at 1500 g. The sperm-free seminal plasma was stored at −20 °C until NGF purification. Then, seminal plasma was loaded into a type 1 macro-prep ceramic hydroxylapatite column (1 cm × 10 cm, 40 µm, Bio-Rad Laboratories, Hercules, CA, USA) equilibrated with 10 mM sodium phosphate (pH 6.8). An eluted fraction showing a major protein on SDS PAGE was concentrated in PBS (pH 7.4) using a 5 kDa cutoff membrane filter device (Vivaspin; Sartorius, Göttingen, Germany) and subsequently loaded onto a gel filtration column (SEC, hi Prep 26/60 Sephacryl S-100; Amersham Laboratories, Piscataway, NJ, USA). The purification procedure was carried out using fast protein liquid chromatography (FPLC; Amersham Laboratories, Piscataway, NJ, USA). Elution was performed isocratically using PBS at pH 7.4.

### 2.2. Animals, Synchronization Protocol, Treatments, and Ultrasonography

**Figure 1:**
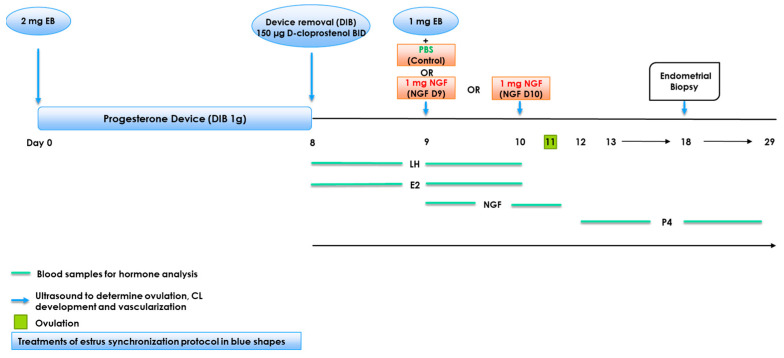
Experimental design of estrus synchronization protocol, treatments, and blood collection.

This study was conducted at the Vista Alegre Dairy Research Station of the UACH. The animals had ad libitum access to ray grass pasture, pellets, and water. From a herd of 45 Holstein-Frisian heifers, a total of 24 cycling heifers, aged 15–17 months old and weighing 328.5 ± 1.1 kg, were chosen based on the detection of a CL by transrectal ultrasonography using a 7.5 MHz linear-array transducer (MyLab30 VET; Esaote Benelux B.V., Maastricht, Netherlands). Heifers were estrus-synchronized using a protocol previously described elsewhere [16,17]. In brief, all heifers received 2 mg of EB IM plus an intravaginal P4 device (1.0 g of P4, DIB^®^, Zoetis, Chile, day = 0). On day 8, the device was removed and heifers received an IM dose of 150 µg of D-cloprostenol (Boviprost^®^, Anasac, Chile). On day 9, 1 mg of EB was given IM and heifers (*n* = 8 per group) were randomly treated with either an IM injection of: (i) PBS (control), (ii) 1 mg of purified NGF at the time of EB administration (NGF D9), or (iii) 1 mg of purified NGF 32 h after EB administration (NGF D10). Ovaries were examined daily by B-Mode ultrasonography from DIB removal to ovulation (the disappearance of the dominant follicle) and then every other day from day 2 (chronological day 12; see Figure 1) to day 18 after ovulation to determine the CL diameter, while power doppler was used on the same days to determine the CL vascularization area. The experimental design and chronological administration of treatments and procedures are shown in Figure 1.

For analysis of Power Doppler images, three distinct images were obtained of each CL, and the maximum blood flow area was calculated using the software Image J (National Institute of Health (NIH), Bethesda, MD, USA) as described elsewhere [10,18].

### 2.3. Blood Sampling and E2, LH, and NGF Assays

A catheter (ID 1.0, OD 1.5 mm) was placed into the jugular vein one day before the DIB removal and blood samples were drawn as shown in Figure 1. Briefly, samples were collected at 12 h intervals from DIB removal and then every 4 h from the second EB administration (t = 0 h) until 48 h for E2 and LH. Samples for NGF assay were collected every 4 h from the second EB administration (t = 0 h) to 52 h in the non-treated group (control), from t = 0 h to 20 h in the NGF D9 group, and from t = 32 h to 52 h in the NGF D10. Blood samples were withdrawn and centrifuged at 2000× *g* for 15 min, and the plasma obtained was stored at −80 °C until hormone determination. 

The plasma E2 concentration was assessed by the solid-phase radioimmunoassay kit, Pantex Estradiol 100 µL direct 125I (cat. 174.1; Pantex, Santa Monica, CA, USA), as previously described [10,13]. The intra-assay coefficient of variation was 1.4–4.3%, the minimum detectable limit was 10 pg/mL, the coefficient of variation of internal standard was <2.45%, and the limit of quantification observed for the assay was 7.92 pg/mL. 

The plasma LH concentration was assessed by a double-antibody radioimmunoassay using an ovine radio-iodinated LH (LER 1374-A), ovine antiserum CSU-204, and ovine LH standard oLH-S25 (provided by NIADDK, Bethesda, MD, USA) in 200 µL duplicates, as previously described [10,19]. The inter-assay coefficient of variation was 0.76–5.47%, the intra-assay coefficient of variation was 0.54–4.5%, the coefficient of variation of internal standard was <3.05%, the minimum detectable limit was 0.07 ng/mL, and the limit of quantification observed for the assay was 0.10 ng/mL.

The plasma beta-NGF concentration was assessed using an enzyme-linked immunoassay (ELISA Duoset cat. DY256; R&D Systems, Inc., Minneapolis, MN, USA) developed to detect human beta-NGF, which was previously validated for the detection of llama beta-NGF [20]. The upper and lower limits of detection were 2000 and 31.3 pg/mL, respectively. The samples were processed and measured in duplicate according to the protocol recommended by the manufacturer. The plasma NGF content was measured in blood samples collected every 4 h. Samples above the limit of detection were diluted (1:2 or 1:4) in blocking buffer and processed according to the protocol recommended by the manufacturer.

Blood samples were collected from the jugular vein every other day, from day 0 to 18 after ovulation, and plasma was obtained as described above. The plasma P4 concentration was assessed with a solid-phase radioimmunoassay kit (PROG-RIA-CT, KIP1458; DIASource ImmunoAssays SA, Louvain-la-Neuve, Belgium), as reported previously [10,14,21]. The intra-assay coefficient of variation was 0.27–3.77%, the coefficient of variation of internal standard was <3.55%, the minimum detectable limit was 0.05 ng/mL, and the limit of quantification observed for the assay was 0.45 ng/mL.

### 2.4. Uterine Biopsy and Gene Expression

Endometrial tissue was collected from a subgroup of heifers (*n* = 4 per group) at 7 days after ovulation from the greater curvature of the ipsilateral uterine horn to the ovary bearing the CL using Jackson uterine biopsy forceps as described previously [22]. In brief, the forceps were covered with a sanitary sheath and, once past the cervix, the tip of the forceps were directed to the greater curvature, where endometrial tissue was collected by closing the instrument jaw. The tissue was placed in a microcentrifuge tube, frozen in liquid nitrogen, and then stored at −80 °C until RNA extraction.

Total RNA was extracted from the endometrium using Trizol (Invitrogen Life Technologies, Carlsbad, CA, USA) according to the manufacturer’s recommendations. The purity of the samples was analyzed using a Nanodrop 1000 (Thermo Fisher Scientific, Inc., Wilmington, DE, USA). One microgram of total RNA was converted to complementary DNA (cDNA) using the kit, AffinityScript Q-PCR cDNA Synthesis (Agilent Technologies, Inc., Santa Clara, CA, USA), and Oligo-dT as per the manufacturer’s instructions. Brilliant SYBR Green QPCR Master Mix (Agilent Technologies, Inc., Santa Clara, CA, USA) was used to detect and quantitate transcripts. Samples were run in triplicate and amplified in a QuantStudio 3 RT-PCR (Applied Biosystems, Foster City, CA, USA) thermocycler using the following thermal cycle conditions: one cycle at 95 °C for 3 min, 40 cycles at 95 °C for 5 s, 60 °C for 30 s, and 72 °C for 1 min. The coefficient of variation between samples ranged from 0.03 to 0.1, depending on the treatment. For each sample, the cycle threshold values for the assayed transcripts were normalized for the total input cDNA (10 ng) concentrations using cycle threshold values for transcripts for the “normalizer” housekeeping gene, β-actin (*ACTB*). Data were analyzed using the thermocycler-associated software, QuantStudio Design and Analysis v1.4 (Applied Biosystems, Foster City, CA, USA). The normalized values in each experiment were compared to a control treatment to determine the relative increase in the amount of transcripts. The target genes assessed were P4 receptor (*PGR*) and amino acid-related transport genes, such as Solute Carrier Family 6 member 1 (*SLC6A1*), Solute Carrier Family 6 member 14 (*SLC6A14*), and Lipoprotein Lipase (*LPL*) related to the histotroph composition. Primer sequences of candidate genes for Q-RT-PCR were elaborated as previously described [23,24]. The accession number of genes and the expected size of amplified products are shown in Table 1. Primer sets for transcript amplification were used at a final concentration of 250 nM each. Data were analyzed using the thermocycler-associated software.

### 2.5. Effect of NGF on 30-Day Embryo Survival—Field Study

The study was conducted on a commercial dairy farm in the province of Rio Bueno, Region de los Rios. The animals had ad libitum access to ray grass pasture, pellets, and water. From a herd of 500 heifers, a total of 450 Holstein-Frisian cycling heifers, aged 14–16 months old and weighing 330.3 ± 0.16 kg, were selected based on the presence of a CL detected with transrectal ultrasonography using a 7.5 MHz linear-array transducer (MyLab30 VET; Esaote Benelux B.V., Maastricht, Netherlands). The heifers were synchronized and treated as described above, but all groups were fixed-timed AI with sexed semen at 48–52 h after DIB removal by a single technician. Pregnancy diagnoses were recorded at day 30 post-insemination by transrectal ultrasonography.

### 2.6. Statistical Analysis

Proportional data (ovulation rate) and non-serial data (i.e., size of preovulatory follicle, CL diameter, and interval to ovulation) were analyzed using Fisher’s exact test and the one-way analysis of variance (ANOVA), respectively. The data for LH, E2, and NGF were centralized to the day of the second EB administration. The data for CL diameter, area of CL vascularization, and P4 concentration profiles were centralized to the day of ovulation (day 0). Serial data (i.e., CL diameter profile, CL vascularization, LH, E2, P4, and NGF concentration profiles) were analyzed with the one-way ANOVA for repeated measures using the PROC MIXED procedure, which considered treatment, day, and their interactions, followed by Tukey’s multiple comparisons test post hoc. The data for gene expression were analyzed with the one-way ANOVA, and the Dunnett test was used to compare the gene expression of all treatments with the control group. Pregnancy rates at day 30 were analyzed using the Chi-square. All data are reported as mean ± SEM and the statistical significance was set at *p* ≤ 0.05. Analyses were performed using the Statistical Analysis System software package, SAS Learning Edition, version 4.1 (SAS Institute, Inc., Cary, NC, USA).

## 3. Results

The size of the preovulatory follicle, interval from the second EB administration to ovulation, ovulation rate, day of the first detection of the CL, CL diameter at day 8, and maximum CL diameter did not show differences among groups (Table 2).

### 3.1. Bovine Plasma NGF Kinetics

**Figure 2:**
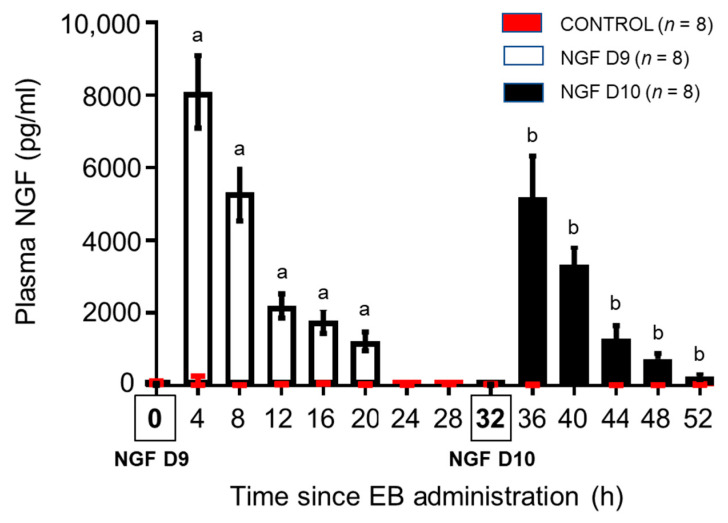
Kinetics of plasma NGF in estrus-synchronized heifers treated with PBS (control), NGF D9, or NGF D10. Data are centralized to the day of second EB administration. Values are mean ± SEM; different superscripts indicate post hoc significant differences between corresponding time points of NGF-treated groups at *p* < 0.05.

After IM injections, plasma NGF concentrations were maximal in both NGF D9 (8094.6 ± 353.9 pg/mL) and NGF D10 (5192.6 ± 401.3 pg/mL) groups at 4 h after administration, and subsequent data analysis revealed a significant (*p* < 0.05) higher content in the NGF D9 than the NGF D10 group at all corresponding time points (*p* < 0.05; Figure 2). The NGF content progressively decayed, but remained above basal levels after 20 h of NGF administration in both the NGF D9 (1217.8 ± 93.0) and the NGF D10 (250.5 ± 31.5) groups.

### 3.2. Effect of NGF on E2 and LH Concentrations

**Figure 3:**
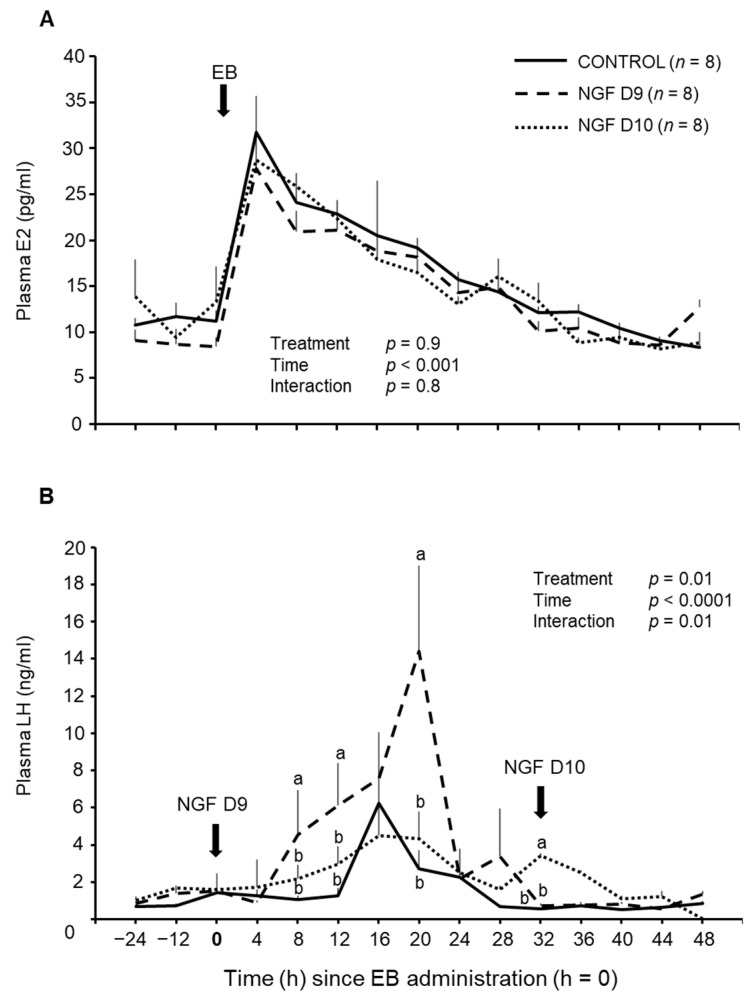
Plasma (**A**) E2 and (**B**) LH concentrations in heifers synchronized with an E2/P4-based, timed AI protocol and treated with PBS (control), NGF D9, or NGF D10. Data are centralized to the day of second EB administration. Values are mean ± SEM; values with different superscripts between groups are different (*p* < 0.01).

The maximum plasma E2 concentration was observed at 4 h after IM treatment with the second EB, without significant differences among groups. The plasma E2 changed over time (*p* < 0.001), but there was no significant treatment effect or interaction (Figure 3A).

Systemic administration of NGF induced changes in LH release patterns. Heifers treated with NGF at the time of the second EB administration (NGF D9) exhibited a greater preovulatory LH surge, with double the plasma LH concentration compared to the NGF D10 and control groups (Figure 3B). Mean values of LH concentration were higher in heifers in the NGF D9 group than in the NGF D10 and control groups at 8 h (*p* < 0.05), 12 h (*p* < 0.01), and 20 h (*p* < 0.01) after the second EB administration (t = 0 h; Figure 3B). The administration of NGF 32 h after the second EB administration (NGF D10) resulted in a significant increase in LH concentration that remained elevated, compared to the NGF D9 and control groups, which had returned to basal LH content by that time point.

### 3.3. Effect of NGF on CL Vascular Area and P4 Production

**Figure 4:**
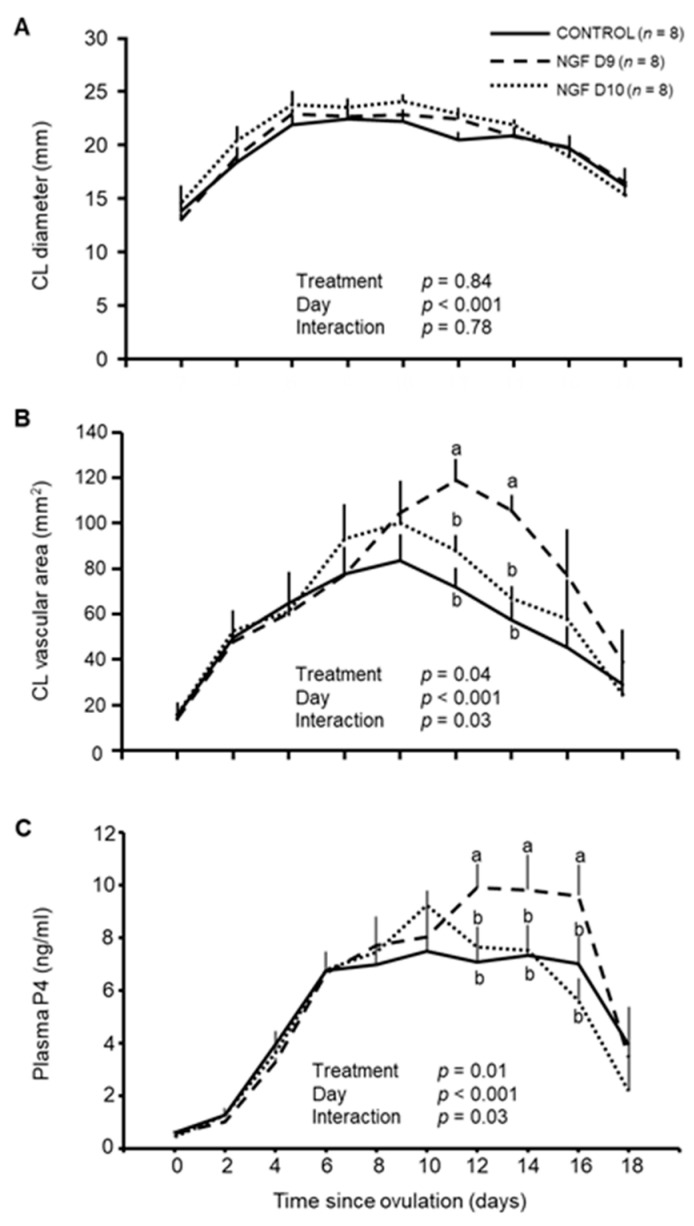
Effect of NGF on luteal function on estrus-synchronized heifers. (**A**) Corpus luteum (CL) diameter profile, (**B**) area of CL vascularization, and (**C**) plasma P4 in heifers treated with PBS (control), NGF D9, or NGF D10. Values are mean ± SEM; values with different superscripts between groups are different (*p* < 0.01).

**Figure 5:**
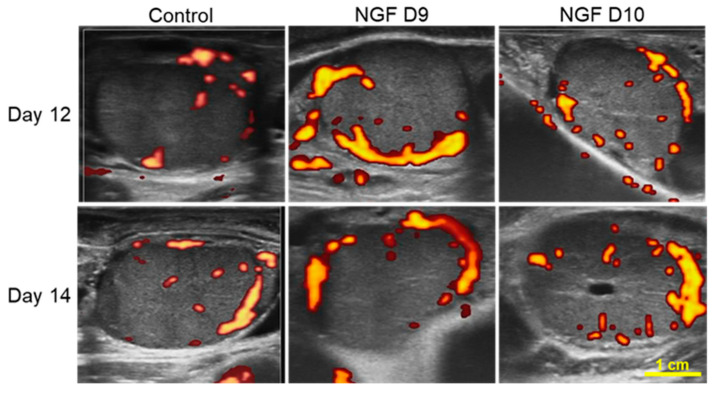
Representative corpora lutea (CL) obtained with Power Doppler ultrasonography, showing the maximum blood flow areas in heifers over the follow-up days.

There was an effect (*p* < 0.001) in the day-to-day diameter profile of the CL without differences among groups (Figure 4A). There was a treatment effect in the CL vascular area (*p* = 0.04) which was greater in the NGF D9 group (*p* < 0.01) than in the NGF D10 and control groups on days 12 and 14 after ovulation (Figure 4B and Figure 5). Plasma P4 concentrations revealed a treatment effect (*p* = 0.01); this was higher in the NGF D9 group than in the NGF D10 and control groups on day 12 (*p* < 0.01), day 14 (*p* < 0.01), and day 16 (*p* < 0.05) after ovulation (Figure 4C).

### 3.4. Endometrial Gene Expression

**Figure 6:**
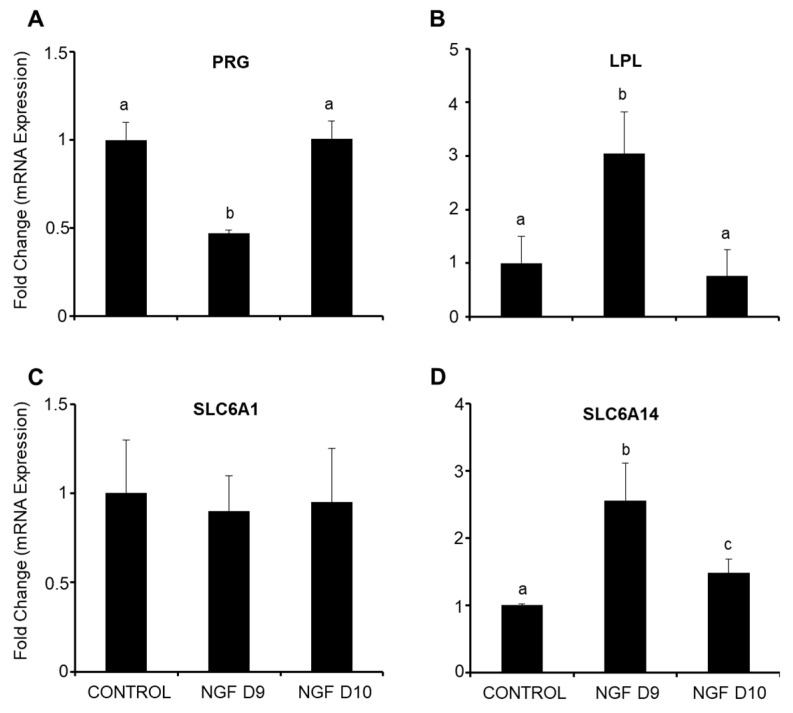
Relative mRNA transcript abundance of (**A**) progesterone receptor (*PGR*), (**B**) Lipoprotein Lipase (*LPL*), (**C**) Solute Carrier Family 6 Member 1 (*SLC6A1*), and (**D**) Solute Carrier Family 6 Member 14 (*SLC6A14*) in estrus-synchronized heifers and treated with PBS (control; *n* = 4), NGF D9 (*n* = 4), or NGF D10 (*n* = 4). Superscripts indicate significant differences (*p* < 0.01) between control and treatment groups.

A significant (*p* < 0.01) downregulation of the mRNA abundance of the *PGR* was detected in heifers of the NGF D9 group, but not in the NGF D10 group. A significant (*p* < 0.01) upregulation in the mRNA abundance of *LPL* and *SLC6A14* was detected in heifers of the NGF D9 group; while the NGF D10 group exhibited a mild but significant upregulation of the latter gene. No changes in the mRNA abundance of *SLC6A1* were detected in any NGF treated groups (Figure 6).

### 3.5. Effect of NGF on Conception Day on Day 30

Three heifers (two from the NGF D9 group and one from the NGF D10 group) were discarded from this study because they lost their P4 devices and were not AI. Ultrasonography testing at day 30 showed that heifers from the NGF D9 and NGF D10 groups exhibited 10% (69/148; 46.6%) and 6% (60/149; 40.2%) higher pregnancy rates, respectively (*p* = 0.2), than control heifers (55/150; 36.6%). The combined NGF group analysis revealed a significant effect of NGF versus non-treated animals (*p* < 0.001).

## 4. Discussion

This study aimed to investigate the effect of beta-NGF administration before and after an EB-induced preovulatory LH release in a fixed-time AI protocol. We found that llama NGF given systemically at the time of the second EB administration in a synchronized protocol results in enhanced LH release, CL vascularization, and P4 production. In addition, we detected changes in the expression of transcripts related to uterine receptivity and histotroph production. After 30 days, heifers receiving NGF had higher conception rates than non-treated animals, and this effect was greater in heifers receiving NGF together with EB.

The most important feature during the periovulatory period is that follicular steroidogenesis shifts from predominantly E2/androgen to P4 production, which is mediated by the action of the preovulatory LH surge. Although the addition of NGF to a primary culture of bovine gonadotroph cells induces an LH release in a dose-dependent manner [25], systemic administration of NGF fails to induce an LH surge in prepubertal or mature heifers [7]. However, heifers subjected to an estrus-synchronization protocol, and ovulated using GnRH plus bovine-purified NGF, exhibited an increased LH release (5.7 ng/mL) compared to non-treated animals (3.3 ng/mL) [12]. Here, we report that the LH surge in the NGF D9 group doubled the concentration quantified in the NGF D10 and control groups, which is consistent with the effect described by Stewart et al. [12], believed to be mediated by a synergistic effect of NGF and GnRH on LH released by the pituitary gland. In our study, administration of EB triggered the endogenous release of GnRH, suggesting potential action of NGF at the hypothalamus, as evidence suggests in South American camelids [3,26].

In the present study, we report the serum kinetics of NGF in cattle, showing elevated concentrations 4 h after administration of a dose of 1 mg, and this was greater in animals given NGF immediately after EB administration (NGF D9), which also exhibited a greater LH peak. A relationship between NGF and LH levels has been previously described in llamas, where the concentration of NGF in the blood was positively correlated with LH plasma content [20]. It is unknown why NGF D10 animals had lower serum NGF content, but it seems unlikely that this fully accounts for the blunted LH response to NGF that was, indeed, prolonged after NGF injection. Potential factors may be related to a late NGF administration regarding the hormonal status in these animals, which might affect the binding of NGF in other tissues, thereby decreasing the bioavailability of this hormone. In line with this finding, the LH surge has been reported to upregulate the expression of the high-affinity NGF receptor, TrkA, in ovarian tissue [27], which possibly enhanced the uptake of NGF from the blood in the NGF D10 group, given NGF after the estimated LH surge using the estrus-synchronization protocol described in [16]. Inversely, estrogens are reported to downregulate TrkA receptors in neural tissue [28], which may have caused the higher plasma NGF content as observed in the NGF D9 group (heifers given NGF together with the second EB administration). Finally, we cannot rule out the possibility of endogenous NGF synthesis as is reported to occur in uterine tissue in rabbits [29].

Tanco et al. [7] demonstrated a luteotrophic effect of NGF in crossbreed Hereford heifers treated with purified seminal plasma NGF, in which increases in CL size and P4 release were detected in comparison with non-treated females. A follow-up study [8], in which heifers received a dose of 25 mg IM of porcine LH to induce ovulation, followed by 12 mL bovine seminal plasma given 4 h after ovulation, showed that plasma P4 concentrations tended to be higher than those of untreated animals. Moreover, heifers given NGF 24 h after EB, coinciding with the LH peak, reached the maximum CL vascular area and plasma P4 concentration on day 9 and day 6 after ovulation, respectively [10]. In the present study, a significant effect of NGF on CL vascularization and P4 production was detected from day 12 after ovulation, when NGF was given together with EB (in the NGF D9 group) but not after the LH peak (in the NGF D10 group). Similar results regarding P4 production were previously reported by Stewart et al. [9] when Angus cows were synchronized using 7-day CO-Synch/CIDR and randomly allocated to NGF or control groups; treated cows showed an increase in P4 concentration compared to control animals from day 10 to 19. Thus, it seems that the timing of NGF administration is crucial for the development of the NGF-stimulated luteotrophic effect, and is possible that maximal luteotrophic action requires prior early ovarian action that primes the tissue for the subsequent effects of the preovulatory LH.

Recent studies conducted in llamas [13,14] have also reported a local effect of NGF at the ovaries that could potentiate P4 production by enhancing the expression of enzymes involved in steroid synthesis. Consistently, a study conducted by Stewart et al. [12] with Holstein heifers showed that administration of NGF plus GnRH results in an increased size of preovulatory follicles without changes in CL vascularization but with higher P4 concentrations. This may be explained by a greater number of small luteal cells and upregulation of transcripts related to the steroidogenic acute regulatory protein (STAR) and the cytochrome P450 family 11 (CYP11A1) that are involved in P4 production. Furthermore, the high-affinity TrkA NGF receptor has been recently detected in the granulosa cells of preovulatory follicles and the luteal cells of CL in bovines [30].

Uterine receptivity can be defined as a physiological state of the uterus when conceptus growth and implantation for establishment of pregnancy is possible [31]. Regardless of the complex understanding of the gene expression pattern reported in different transcriptome studies, the early downregulation of the endometrial P4 receptor during the luteal phase is a mandatory condition for uterine receptivity in ruminants for both pregnant and non-pregnant females [32]. Consistently, we found a decrease in *PGR* mRNA in the NGF D9 group, together with upregulation of *LPL* and *SLC6A14* transcripts, which have key roles in triglyceride metabolism and amino acid transport in the formation of the histotroph. In line with this, it has been well documented [24,33] that P4 receptor downregulation induces a change in the expression of genes related to histotroph composition. Furthermore, low P4 concentration in Nelore cows (Bos indicus) has been linked to reduced expression of some genes related to amino acid transporters, and also reduced amino acids content in uterine washings, including valine and cysteine, seven days after GnRH-induced ovulation [23].

Few studies have addressed whether the effects of NGF on P4 and amino acids transporters lead to an improvement in pregnancy rates. Stewart et al. [9] described a 16% higher pregnancy rate (75 vs. 59% in NGF and control groups, respectively) at day 28 after fixed-timed AI in Angus cows treated with 296 ug of NGF. Moreover, NGF-treated cows showed a greater concentration of pregnancy-specific protein B (PSPB) at day 24 post-AI, and upregulation of transcripts (i.e., ISG15, MX2) associated with maternal recognition of pregnancy. Conversely, in lactating dairy cows, pregnancy rates did not differ between NGF-treated and non-treated females, 37 days after AI [11]. Here, the pregnancy rate was numerically higher in those heifers given NGF either at day 9 (46.6%) or day 10 (40.2%) than in non-treated females (36.6%). Although our crude pregnancy rates (in all groups) were lower than previously described [9], in this study, heifers were AI using sexed semen, so results are in the percentage range reported in other studies [34,35]. It is likely that the action of NGF may be affected by different factors, such as beef or dairy breeds, heifers or cows, the NGF dose administered, and perhaps the source of the NGF (i.e., homologous or heterologous). More research is needed to fully understand the mechanism of action of NGF on bovine reproduction and whether it is economically justifiable to include it in AI protocols.

The NGF present in the bovine seminal plasma regulates sperm physiology, ovarian function, and markers of conceptus development [7,8,9,36], playing a key role in modulating fertility. This has special relevance in the dairy cattle industry, as AI is extensively used to select desirable traits in milk production. Studies on bull semen cryopreservation have focused on developing the most suitable semen-freezing protocols and extenders to accomplish a fertilizable dose; however, the use of extenders results in the dilution of seminal plasma proteins, including beta-NGF, underestimating their biological role in fertility. 

## 5. Conclusions

Systemic administration of heterologous beta-NGF during the early periovulatory stage results in enhanced LH release, CL vascularization, and circulating P4 concentration. It is likely that the higher P4 output in response to the early NGF administration (in the NGF D9 group) led to enhanced gene expression of key transcripts related to uterine receptivity and histotroph composition, including *PGR*, *LPL,* and *SLC6A14,* which resulted in the enhanced pregnancy rate 30 days after AI with sexed semen. 

## Figures and Tables

**Table 1:** Gene accession number, Entrez gene symbol, primer sequences (F: forward, R: reverse), and amplicon size for all genes used for Q-RT-PCR.

Accession No.	Entrez Gene Symbol	Primer Sequence (5′-3′)	Amplicon Size (bp)
NM_001205356.1	*PGR*	F: GAGAGCTCATCAAGGCAATTGG R: CACCATCCCTGCCAATATCTTG	199
NM_001077836.1	*SLC6A1*	F: GTGTCTCCATTTCCTGGTTT R: GCACAGCACTGAAGATGAA	150
NM_001098461.1	*SLC6A14*	F: GATGCTGCCACACAGATATT R: TAGCAAATCCAGCGAACAC	154
NM_001075120.1	*LPL*	F: CAGGTCGAAGTATCGGAATCCA R: GAAAGTGCCTCCGTTAGGGTAAA	67
XM_027528015	*ACTB*	F: GGATGAGGCTCAGAGCAAGAGA R: TCGTCCCAGTTGGTGACGAT	77

**Table 2:** Follicular and luteal parameters, interval to ovulation (h), and ovulation rate (%) following systemic NGF administration during the preovulatory stage in dairy heifers (n = 8 per group, mean ± SEM).

	Control	NGF D9	NGF D10	*p*-Value
Follicle size at DIB removal (mm)	13.2 ± 1.1	11.5 ± 0.5	12.8 ± 1.6	*p* = 0.8
Follicle size at Day 9 of synchronization protocol (mm)	15.4 ± 1.6	13.9 ± 0.6	14.4 ± 1.0	*p* = 0.6
Follicle size at Day 10 of synchronization protocol (mm)	16.4 ± 1.4	15.6 ± 0.5	15.4 ± 0.7	*p* = 0.7
Interval from second EB administration to ovulation (h)	45.0 ± 3.0	46.5 ± 2.7	43.5 ± 3.9	*p* = 0.9
Ovulation rate (%)	8/8 (100%)	8/8 (100%)	8/8 (100%)	*p* = 1.0
CL diameter (mm)	24.0 ± 1.3	23.0 ± 0.5	24.4 ± 1.2	*p* = 0.7
Day of maximum CL diameter (mm)	6.7 ± 0.4	6.2 ± 0.4	7.0 ± 0.4	*p* = 0.8
CL diameter at Day 8 after ovulation	22.7 ± 1.6	22.6 ± 0.5	24.1 ± 1.0	*p* = 0.9

## Data Availability

The datasets generated during the present study are available from M.H.R. upon reasonable request.
